# Measuring Well-Being: Trial of the Neighbourhood Thriving Scale for Social Well-Being Among Pro-Social Individuals

**DOI:** 10.1007/s42413-020-00067-6

**Published:** 2020-06-10

**Authors:** Cathy Baldwin, Penny Vincent, Jamie Anderson, Patrick Rawstorne

**Affiliations:** 1 Institute of Health and Wellbeing, University of Glasgow, Glasgow, UK; 2 University of Oxford, Oxford, UK; 3 Oxford Brookes University, Oxford, UK; 4 University of Staffordshire, Stoke-on-Trent, UK; 5 University of Manchester, Manchester, UK; 6 University of New South Wales, Sydney, Australia

**Keywords:** Subjective well-being, Community, Measurement scale, Social epidemiology, Psychology, Stoke-on-Trent

## Abstract

We report on a trial of the *neighbourhood thriving framework* (NTF), a conceptual framework from psychology and social science for measuring collective subjective social well-being. It combines the notions of feeling good and functioning effectively in a neighbourhood social environment in an indicator set of 15 conceptual dimensions. An online questionnaire was used to measure *neighbourhood thriving* (NT) among 212 pro-social volunteers involved in revitalising neighbourhoods in the UK city of Stoke-on-Trent between May and October 2018. Exploratory factor analysis revealed 11 factors that made conceptual sense including three social epidemiological pathways to well-being, networks, participation and pro-social behaviours, and four criteria for *flourishing* societies, autonomous citizenship, safety, cohesive communities and resilience. The 11 sub-scales of NT showed satisfactory internal consistency reliability and preliminary evidence of construct validity. The sub-scales were used tentatively to examine NT among the volunteer sample, which showed the highest sub-scale score for *Positive Regard* and the lowest score for *Celebration*. Different levels of NT were observed among the community, with age and income positively associated with higher levels of NT. Further validation work is needed before the NT scales can be used with confidence. Validated scales offer potential benefits including: measuring NT pre- and -post project implementation; establishing which dimensions of NT are, and are not, working well in a community and need strengthening through further initiatives, and establishing which specific groups of people are experiencing lower levels of NT and designing projects that meet their needs.

**Electronic supplementary material:**

The online version of this article (10.1007/s42413-020-00067-6) contains supplementary material, which is available to authorized users.

## Introduction: Neighbourhood Flourishing

We report on a trial of a conceptual framework measuring social well-being called the ‘neighbourhood thriving framework’ (originally outlined under the name ‘neighbourhood flourishing framework’ in an earlier paper by authors Anderson and Baldwin (2017) in the English city of Stoke-on-Trent. The neighbourhood thriving framework is based on an original framework called the ‘neighbourhood flourishing framework’ (NFF) that was designed to measure the effects of urban design on neighbourhood flourishing (NF). It thus emphasised a place-based conceptualisation of ‘neighbourhood’ in daily life. The current paper tests the 14 original dimensions and 1 new one (affection) that we added on a sample of volunteers from city-wide networks intentionally engaged in prosocial activities at a neighbourhood level. In a conceptual departure from the original NFF framework, this specific target group was selected due to there being three well-established social epidemiological causal pathways to well-being anchored in pro-social social structures and action: networks, participation and pro-social behaviours. Pro-social action in local communities is urgently needed to combat dangerous global crises such as climate change and pandemics that affect many communities around the world. In using our adapted version of the NFF (known here as the *neighbourhood thriving framework* for differential purposes) to collect data from pro-social volunteers, we sought to perform exploratory factor analysis, a statistical analysis technique, on the data to see to what extent the resulting factor structure reflected these three pathways.

## Original Framework

The NFF was devised by Anderson. It comprised a preliminary indicator set of 14 features centred on the combined notion of *social feelings* and *functionings* at the group, not individual level, in relation to geographic or non-spatial communities. These were derived from Huppert et al.’s (2006) individual well-being framework, based on current psychological theory, and developed for the European Social Survey (ESS). It distinguishes interpersonal features (e.g. receiving help from others) from personal feelings (e.g. I feel happy). Anderson introduced four additional features.

The ESS framework measured well-being among individuals. It evolved from developments in psychology where individual subjective well-being is conceived of as split between momentary experiences of pleasure or positive emotions (hedonic well-being) *and* more important, long-term and active processes of ‘well-doing’ (Aristotle’s eudaimonia) (see, e.g. Keyes 2002; Ryan and Deci 2001; Huppert and So 2013). Hedonia is theorised as a passive process of attaining the state of feeling good. Eudaimonia is said to involve ‘being holistically engaged, being challenged and exerting effort’ (Waterman 1993, cited in Anderson and Baldwin 2017, p. 316), which meets needs rooted in human nature, such as the realisation of potential (Waterman 1993; Ryan et al. 2008, cited in Anderson and Baldwin 2017, p. 316).

Huppert and So (2013) combined these two types of wellbeing - feeling good (hedonic well-being) and functioning effectively (eudaimonic well-being) - into an operational definition of individual mental well-being increasingly referred to as *flourishing*. They captured each form through an objective list of *feelings* and *functionings.* When compared with the results of a single survey question about ‘life satisfaction’, a traditional survey proxy for well-being, they found that the single measure lost much information and confirmed subjective well-being as a multi-dimensional construct (p. 839).

Whilst there are other indicator sets that combine hedonia and eudaimonia, they are not designed to measure neighbourhood-based flourishing, as shaped by the particular social experiences that people accrue by living in a geographically defined neighbourhood. The most widely used measures include the Personally Expressive Activities Questionnaire Standard form (PEAQ-S) (Waterman 1993), the Orientations to Happiness Scale (OTH) (Peterson et al. 2005), The Satisfaction with Life Scale (Diener et al. 1985), the Scales of Psychological Well-being (Ryff and Keyes 1995), and the Hedonic and Eudaimonic Motives for Activities (Huta and Ryan 2010). While these scales provide general measures of hedonia and eudaimonia they do not indicate the sources of well-being and happiness and nor are they linked to neighbourhoods and local communities.

## Measuring Well-Being on the Group/Social Level

With the exception of one item (positive relationships), Huppert and So’s scale did not address social well-being – well-being at the group or community level, i.e. how the individual responds to experiences of the social environment which can affect their health (Larson 1993; Keyes 1998). As individual local communities around the world face increasingly more frequent challenges that affect all members and put a strain on their daily social environment, (e.g. climate change, pandemics, and socio-economic crises), many people’s well-being will be affected. Well-being is a core component of group social resilience (Zautra et al. 2008), a quality that communities can collectively cultivate that allows them to cope with, and respond effectively to these shock experiences. Other essential components of social resilience are social capital (participation in networks for collective benefits) and social cohesion (cohesive relationships between social group/network members) (Baldwin and King 2018). Whilst the individual members of a population may be feeling good and functioning effectively on an individual (personal) level, the same people might respond weakly at a community-level to a big challenge, (e.g. a natural disaster) (Anderson and Baldwin 2017). Strong communities experiencing high levels of well-being are more likely to develop the ‘adaptive capacity’ (Berkes and Ross 2013) to respond resiliently to challenges (Baldwin and King 2018). Therefore, being able to measure (and maintain) a type of aggregated individual well-being that is grounded in shared social experiences (i.e. social well-being or *flourishing*), will be a key aspect of assessing and maintain community resilience.

Social health and well-being are recognised as a distinct phenomenon (e.g. Larson 1993; Keyes 1998; Keyes et al. 2007) from individual psychological and emotional well-being (Keyes et al. 2007, p. 100), and physical and mental well-being (World Health Organization 1946/1948). Whilst there is no widely accepted common definition, ‘social health’ (Larson 1993, p. 287) describes the health of society and factors such as the distribution of wealth (McDowell and Newell 1987), or the influence of social phenomena on individuals. The latter has been defined as: ‘that dimension of an individual’s well-being that concerns how he gets along with other people, how other people react to him, and how he interacts with social institutions and societal mores’ (McDowell and Newell 1987, p. 152).

Keyes (1998) proposed a conceptualisation of social well-being that situates an individual within social structures. That person evaluates their situation and personal functioning against social criteria (*italicised*), whereby they appraise themselves in the context of: 1) *integration* (relationship with community/society); 2) *contribution* (their social value and contribution); 3) appraise others: *acceptance* (trust in others); and appraise society/community: 4) *actualisation* (evaluation of society/communities’ potential); and 5) *coherence* (perceived quality and organisation of society/community) (Keyes et al. 2007, p. 100). The original NFF (Anderson’s work) built upon Keyes’ work, and Aristotle’s Eudaimonia to argue that ‘a well-lived life includes the quest for positive social lives, involving meaningful interaction with family, community and wider society’. Going beyond individual psychological functioning, the presence of both *social feelings* (e.g. I feel close to my community) and *social functioning* (e.g. I feel supported by my community) reach beyond perceptions of one’s self and represent a key part of positive social health.

As public health scholars, we were also aware of the broader social epidemiology approach that situates psychological approaches to well-being within an epidemiological model of causal pathways between factors that affect human health and well-being, and health and well-being outcomes. Social epidemiologists (e.g. Kiwachi and Kennedy 1997; Berkman and Glass 2000; Kiwachi and Berkman 2000; Wilkinson and Marmot 2003) charted causal pathways between the individuals’ social experiences of the social environment, encompassing interactions with others within it for different purposes, and personal physical and mental health and well-being status. They explore pathways between individual and group experiences of processes described by sociological concepts such as social integration (as defined by Keyes); social networks - the structures of society/community; social cohesion - the quality of individual and group-level relationships within networks; social capital - the ways in which networks operate to provide members with benefits; the provision of social support - a particular kind of network benefit; *and* individual and group-level health and well-being.

Both psychology and social epidemiology recognise the roles of the individual, others with whom they interact, and the social environment as determinants of social well-being (McDowell and Newell 1987, p. 152; Keyes et al. 2007, p. 100; Berkman and Glass 2000; Kiwachi and Kennedy 1997). The NTF uses proxy indicators, otherwise known as construct scales, which aim to measure the specific feelings and functionings that individuals experience at the interface between individual and social experiences. Psychology focuses on the internal psychological processes that individuals experience during and through such interactions and processes during daily life. Social epidemiology identifies the wider social processes and dynamics operating in broader community, societal and institutional contexts, and evinces concrete links to health and well-being. These approaches are combined in our conceptual framework.

The dimensions of social well-being that we tested in this study, then, are the 14 conceptual dimensions in the NFF and one additional dimension. They are described in Table 1 below. We named this set of conceptual dimensions the *neighbourhood thriving* framework to differentiate it from other *neighbourhood flourishing* trials run by author Anderson. Each dimension was represented in our study by a set of 5–7 proxy indicators – commonly known as ‘items’ in psychology questionnaires (see below). Each item uses a statement for survey respondents to respond to by testing this framework on pro-social people rather than all residents of a neighbourhood, we had the following questions:

1. Did pro-social volunteers’ responses lead to any variations in the dimensions of well-being that emerged when exploratory factor analysis was performed?
2. To what extent did the resulting factor structure reflect the three social epidemiological pathways to well-being: networks, participation and pro-social behaviours?

## Social Epidemiological Pathways/Mechanisms Underpinning Social Well-Being

The neighbourhood is the geographic location of the local social environment, but within this social environment, there is a sub-network of pro-social volunteers aiming to produce pro-social outcomes from their volunteer activity. Each individuals’ experience of volunteering occurs in social networks with fellow volunteers. Equally then, 1) the volunteer network, 2) the wider social environment created through the interaction of people in neighbourhoods during voluntary activity, and 3) the prosocial activity, may all influence respondents’ social well-being outcomes. Accordingly, we adopted the social epidemiological emphasis (Berkman and Kiwachi 2000) on the social health and well-being effects of social networks and civic activity, identifying the three aforementioned possible causal pathways (each a sociological concept with sub-components) to an individual’s positive social well-being matching the position of the volunteers in our study.

### Social Networks

As non-spatialised communities are becoming more common (Anderson and Baldwin 2017, p.314) and volunteers were distributed throughout the city, although acting in neighbourhoods, conceptually, we focused away from geographic neighbourhood-based communities to communities-as-social-networks (see also Berkman and Glass 2000) as a sub-network within the neighbourhood. Berkman and Glass (2000, p. 141–2) used a network approach to understand how the structure and function of social relations and networks influences health outcomes. They drew on the analysis of earlier anthropologists (Barnes 1954; Bott 1957) of the ways that networks ‘cut across traditional kinship, residential and class groups’ (Berkman and Glass 2000, p. 140) to explain the benefits that network members acquired such as jobs and political activity, and emphasises analyses of the structural qualities of the relationships among people in the network, its composition, and the type of resources that were available through it. Social networks are linked to health outcomes through four sub-casual paths: through their functions in providing social support, the social influence of network members on other members, e.g. the provision of information on maintaining good health, social engagement and attachment, and the access that networks provide to resources and material goods (Berkman and Glass 2000, p.143).

### Social Participation and Engagement

Social participation and engagement is a related concept to ‘network’ that builds on its inherent abilities to connect people, and which is influential in social psychology, and the social and political sciences. It is described as ‘a process in which individuals take part in decision making in the institutions, programs, and environments that affect them’ (Heller et al. 1984; Wandersman and Florin 2000, cited in Keyes et al. 2007, p. 98). It involves people participating in a community context in social activities in networks, as shaped by local issues, the geographic location, and local ‘culture, norms, values and institutions’ (Keyes et al. 2007, p. 98). This describes the position of our volunteers.

Contributing to the community through participating and engaging with other participants has been linked to aspirations for life and well-being (Keyes et al. 2007, p. 99–100). Berkman and Glass (2000, p. 146–7) elaborated on an unclear pathway between social engagement and participation and positive health status. They saw participation as the face-to-face ‘enactment of potential ties’ in informal and formal settings where people take on social or occupation roles (e.g. as a community volunteer, as per our study, which can give them feelings of ‘value, belonging and attachment’). Participating in a social role within the network reinforces personal identity (Berkman and Glass 2000, pp.146–7), provides company and sociability, and on the collective level, networks are a key feature of social cohesion, also a determinant of positive health status (Kiwachi and Kennedy 1997). The authors found that ‘contact with friends and family, and participation in voluntary activities’ gives life ‘a sense of coherence, meaningfulness and interdependence’ (p. 147). They linked social participation and engagement indirectly via coherence and identity to improved levels of well-being, and directly various physical health outcomes. The participation and engagement aspect of volunteering could also contribute to our volunteers’ social well-being.

### Pro-Social Behaviours

When people participate in social networks, a third pathway emerges between the actual prosocial behaviours and actions that each individual engages in, and the influence of these on their health and well-being, and that of an aggregation of their fellow community members’. Ryan et al. (2008) have described the eudaimonic component of flourishing as a way of life. They reviewed a number of studies that collectively showed that people who live in a eudaimonic way are more likely to exhibit prosocial behaviour, thus benefiting themselves and other people in their wider community. Individuals who undertake much social and volunteering activity have shown correlations with positive affect (the emotional component of well-being) and negative correlations with depression (Larson 1993, p. 286). In reverse, well-being itself is said to result in prosocial behaviour (Huppert and So 2013, p.838), so there is a hypothesised bidirectional pathway of influence.

Ryan et al. (2008) also found that ‘conditions both within the family and in society more generally contribute toward strengthening versus diminishing the degree to which people live eudaimonic lives’ (Ryan et al. 2008, p139). Our study was able to test out this statement by exploring the extent to which Stoke’s social environment was associated with pro-social activity.

## Devising the Neighbourhood Thriving Framework for Testing on pro-Social Volunteers

The 14 conceptual dimensions from the original NFF framework are mainly an extension of Huppert et al.’s well-being frameworks for the 2006 and 2012 ESS (Huppert et al. 2012). To these, author, Anderson, added three further concepts: safety, participation and celebration. Safety and participation were included in light of Roger’s (2003) substantive development of a social cohesion index and their absence from the ESS model. Celebration overlaps with Roger’s concept of *creativity* (community that encourages imagination and boldness) but also includes the notion of appreciation, and affection. We incorporated an additional dimension, ‘affection’, into the adapted *Neighbourhood Thriving* framework trial because loneliness and isolation are detrimental to mental health, and “friendliness” is a valued quality in UK communities.

**Fig. 1:**
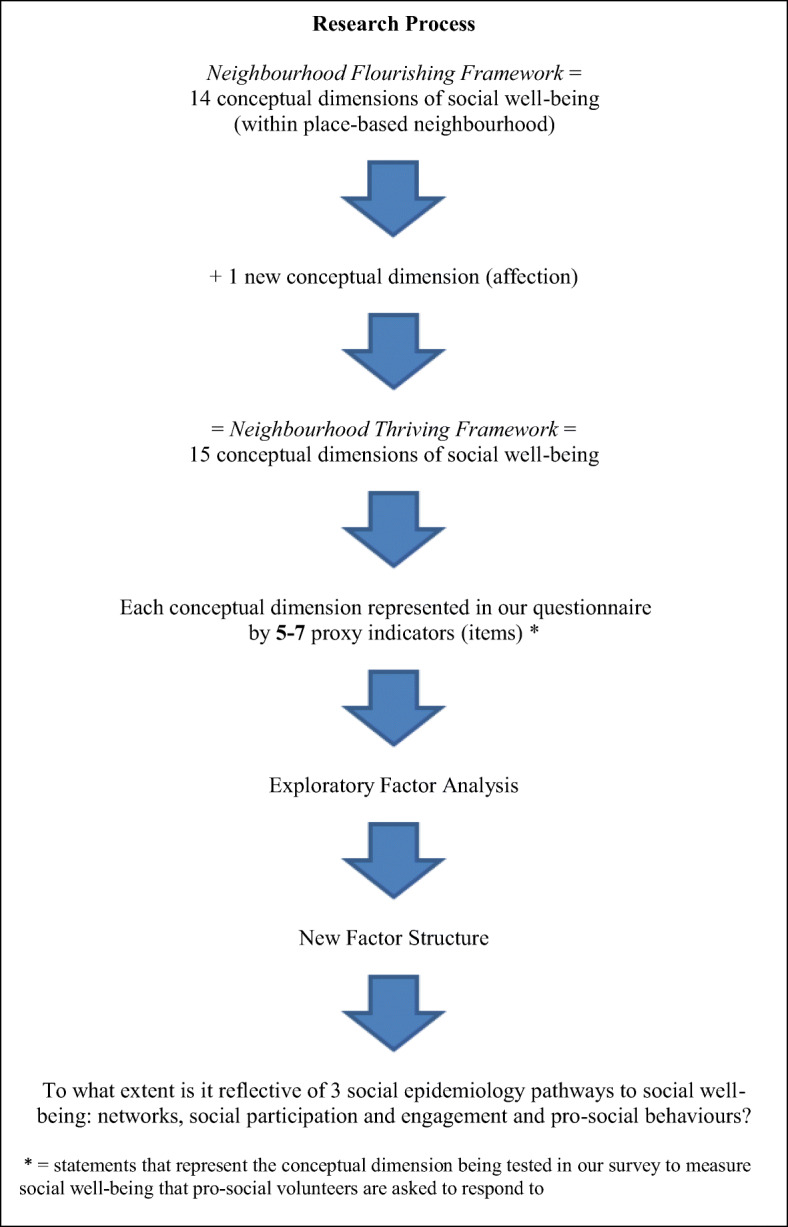
Research Process

Our aim in the present trial was to achieve sub-scales with a minimum of 3 items in each to provide reasonable reliability and validity of each sub-scale. To achieve this, Baldwin and Rawstorne devised 5–7 proxy indicators per dimension (sub-scale) in anticipation of the potential loss of proxy indicators during Exploratory Factor Analysis (EFA) and reliability analysis. Where possible, proxy indicators were adapted from the ESS (2006; 2012), and existing national UK surveys, the *Citizenship Survey* (2010–11; UK Data Archive Study No 7111), *UK Household UK Household Longitudinal Study* (Institute for Social and Economic Research 2014), *ONS Social Capital Indicators Review* (Foxton and Jones 2011) and surveys from environmental psychology (e.g. Williams and Vaske 2003) and social epidemiology (e.g. Cobb 1976). Elsewhere, original proxy indicators were devised through discussion/refinement between the authors, with disagreement rare and discussed until agreement was reached. The survey questionnaire is in the Appendix, and was reviewed by Stoke project leads. Face validity (i.e. proxy indicators appearing to measure what they are intended to measure) and content validity (i.e. proxy indicators measuring the full breadth of each construct) were built into the design of the proxy indicators measuring each sub-scale. A summary of the research process ranging from the utilisation of the *Neighbourhood Flourishing Framework* through to the development of the *Neighbourhood Thriving* scale is depicted in Fig. 1.

## Materials and Methods

The study received ethical approval from the University Research Ethics Committee at Oxford Brookes University, UK, and Human Research Ethics Committee at the University of New South Wales (UNSW), Australia.

### Study Location

We sought volunteers involved in pro-social/community activities at neighbourhood level, via a request circulated to public health authorities/community development organisations in UK cities/regions using our networks and online mailing list *Community Empowerment Evidence Network* CEEN@JISCMAIL.AC.UK Interest was received from Aylesbury, Sheffield, Exeter and Stoke-on-Trent, and South Wales. Stoke-on-Trent (hereafter known as ‘Stoke’) (estimated population of 255,378 in 2017; 88.5% ‘White’ ethnicity) was selected because it is one of the most deprived cities in England – 14th out of 326 local government authorities. Over half the population (53%) live in areas that are among the 20% most deprived in the country (all: Stoke-on-Trent City Council 2011).

We also chose Stoke as people in the city are generally disengaged on civic and organised community levels. Residents demonstrate the lowest participation in volunteering (7.37% - just below the county’s average) and lowest voting rate (54%) in the county (region) of Staffordshire. Social cohesion is challenging. Just 60.8% of people say they believe people from different backgrounds get on – the national average is 76.4%. And 26.9% of people thought they could influence decisions compared to the national average of 28.9%. And 44.3% of people feel that people do not treat each other with respect, with a national average of 31.2% (Staffordshire Community Foundation 2015).

In response, Stoke-on-Trent City Council Public Health Department commissioned two community development projects in 2012 to reduce health inequalities through social action, each run by a separate Community Interest Company (CiC - a social business, with stated community benefit).

- *My Community Matters (MCM)* engages residents in social action in 10 neighbourhoods. In each, community development workers collaborate with residents, community groups, businesses and organisations to set up neighbourhood partnerships. They empower residents to lead community activities, to identify local assets, improve neighbourhoods and inspire further action. http://mcmstoke.org.uk/
- *1000 Lives Network* is a city-wide virtual network that was developed in response to demand from active citizens for opportunities to connect for mutual support to sustain their social action and engage others in volunteering. Motivated residents, civil society and voluntary groups, and paid workers support volunteers and community groups. The Network provides training in skills for community leadership, facilitates resource sharing, supports social action project start-up, and celebrates ‘community champions.’

By 2017, *1000 Lives* and *MCM* were in contact with a minimum of 2000 volunteers, but were unaware of how many participated in both. Keen to gain a baseline measure of participants’ social well-being (NF), they agreed to inform their networks about our online survey hosting the trial, and assist respondents to access it using mobile phones and computers.

### Research Instruments

Development of the *neighbourhood thriving* framework scale for social well-being involved several stages including: item development, piloting and revising item statements, as well as reliability and validity testing (including content validity, face validity, construct validity, and Cronbach’s alpha coefficient internal consistency reliability).

#### Item Development

Item (proxy indicator) development occurred between December 2017 and January 2018. In the statements, we distinguished between the physical locality of a neighbourhood, and its human residents to make it clear which one we were talking about. We phrased this distinction in the questionnaire using terms taken from the two Stoke initiatives:

“By **NEIGHBOURHOOD**, we mean: the buildings and outdoor spaces where you live and/or work where you see local people”.

“By **COMMUNITY**, we mean: the people who live, work, study, volunteer, run services and businesses locally with whom you interact, other than your family”.

This distinction was stated before and mid-way through the 15 dimensions-based sets of statements in the online survey. The premise of our research was that volunteers conducted activities on a face-to-face basis within spatial proximity of each other, not a virtual one, hence the questionnaire did not address online activity. However, in conceptual research terms, there is no guarantee that the human residents of a physical neighbourhood will form any kind of network or social community. It is not uncommon in Northern Europe to find that people do not know or cooperate with their neighbours in any meaningful way. This norm may be changed significantly after the current Covid 19 pandemic which is showing the importance of local cooperation and neighbourly relationships. The original NFF in part sets out to measure what level of shared social experiences occur in any given neighbourhood, and how these impact on residents’ social well-being. The Stoke projects intended to galvanise residents to come together to work collectively on improving their neighbourhoods, and to enhance networks and feelings of social community. With this additional pro-social effort on top of the routine workings of neighbourhoods, how would this impact the final factor structure?

### Instrument Development

The *Stoke-on-Trent Well-being Survey Questionnaire* (SOTWSQ) was constructed for the purpose of collecting data for this trial and hosted by online platform *Qualtrics*: https://www.qualtrics.com/uk/. Respondents were guaranteed anonymity and gave their consent via an online form prior to completion. A downloadable sheet listing the contact details of the authors, community development workers from *1000 Lives* and *MCM*, and local mental health support services for respondents to ask questions or seek support should they experience any discomfort after completing the questionnaire was clearly marked beside the consent form. It was not expected that completing the questionnaire would result in adverse effects due to the gentle nature of questions, and no respondents contacted the team with questions.

After responding to demographic questions, they were asked to answer 15 questions – one relating to each dimension – by responding to 5–7 items per dimension by selecting an answer option for each from a 5-point Likert scale, from 1) Strongly agree, 2) Agree, 3) Neither agree nor disagree, 4) Disagree, and 5) Strongly disagree. The sub-scales are named in Appendix A for reader clarity and have been re-labelled, but were not named or numbered as such in the questionnaire. Some items were inverted at random to mix up the order of positive and negative statements so as to identify response bias (i.e. respondents ticking all answer options down the left- or right-hand side of the page). A full list of the scale items and their sources are provided in Appendix A.

### Sampling Procedures

We opted for a convenience sample of a minimum of 200 volunteers which was not randomised or stratified. Volunteers’ addresses were already known and there was no viable way of randomising their selection as relevant demographic information was unavailable. Instead, the limitations of a convenience sample were accepted, and the following survey inclusion criteria were set for respondents: they should be aged 18 or over, live or work in Stoke, volunteer with *MCM*, *1000 Lives* or another local community action group, and be able to complete the survey online. A pilot trial was run with the first 20 volunteers at a community meeting in Stoke. They completed it on laptops (some with support worker assistance) following an introduction by authors Baldwin and Rawstorne, who visited. Respondents provided verbal feedback on the pilot version of the survey and small changes were made to the wording of survey items for clarity, and incorporated into its final design. No items were deleted. Project workers used their face-to-face contacts, email distribution lists, Facebook pages, Twitter, and community group webpages to inform volunteers about the survey and invite them to take part.

### Data Collection Time-Frame

Online data collection for the full community trial took place in Stoke-on-Trent between May and October 2018. Data collection for the pilot trial ran from late April to early May, and the community trial ran from early May to October 2018. The pilot data was not used in the final analyses, only the data from the trial.

### Data Analyses

Data cleaning was minimal since the instrument was administered via the *Qualtrics* site with skips (routing) built into the programme. Exploratory factor analysis (EFA) using principal axis factoring with oblique rotation was performed on all 85 items, based on the responses of 212 participants who lived and/or worked in Stoke-on-Trent. Cronbach’s coefficient alpha was calculated for each factor to determine the scale internal consistency reliability as well as whether the reliability of each sub-scale could be improved by the deletion of any items. Removal of items from scales was also informed by conceptual considerations. Construct validity was tested in two ways: first, by correlating each sub-construct with other sub-constructs, after making predictions about the direction and strength of each association, and second by: conducting analyses between sub-constructs and other study variables that were expected to show a relationship or difference. Once satisfied that the sub-constructs showed preliminary evidence of reliability and validity, we conducted further analyses of NT in Stoke.

Many of the analyses, including those used to help describe the sample, were examined across two variables of interest: sex of participant and judgement about whether their neighbourhood had got better to live and/or work in over the preceding 2 years.

## Results

### Characteristics of the Sample

A majority of the 212 participants were women (*n* = 144; 69%), and 66 (31%) were men (Table 2). Over one-third of the sample participated in the *MCM*, while 50% were involved in projects other than *MCM* and *1000 lives* (Table 2). In answering whether their neighbourhood had got better to live and/or work in the prior 2 years, 65 people (31.4%) agreed to some extent, 56 (27.1%) were neutral, and 86 (41.5%) disagreed, thinking that their neighbourhood had got worse. The age of participants ranged from 18 to 86 with most between 35 and 64 years. Most participants were living in households alone or with another adult (Table 2), mostly without children at home.

**Table 1:** The 15 conceptual dimensions of *neighbourhood thriving* tested in this study

Feelings	Description
Belonging	Sense of belonging to people in the local area in terms of attachment and identification
Respect	Perception that other local people treat the respondent with respect
Trust	Measures the extent to which respondents expect fairness from, and trust, other local people
Support	Two sub-features: (a) The extent to which people feel they have others who support them. (b) Perception of how much people in the respondent’s local area help each other including all types of support
Safety	How safe respondents feel when they use their neighbourhood in everyday life
Functionings	Description
Reciprocity	A balance between give-and-take or reciprocity in social exchange (i.e. perception of support from locals and providing help and support to them in turn)
Celebration	Two sub-features: the extent to which people feel that their local community values and actively celebrates (a) fellow members and (b) creativity
Engagement	Positive social relationships, that can be intimate or more informal – as in more numerous but more superficial
Autonomy	Residents’ perceptions of their influence over the local area and in shaping community life or activities, free from others people’s control
Resilience	Returning to a previous level of normal functioning, following severe period of hardship
Altruism	Genuinely doing something good for someone, for its own sake and not for personal gain
Contribution	An evaluation of one’s social value. It includes the belief that one is a vital member of society, with something of value to give to the world
Optimism	An individual’s assessment of the future of their local community as a whole
Participation	Realisation of opportunities to be engaged and included locally – through access, cooperation, skill and effort
Affection*	Feelings of being appreciated and welcome in an individuals’ community, based on the experiences of group-level friendliness or positive validation* additional dimension added by lead author

**Table 2:** Participant characteristics (*N* = 212)

		Sex of participant^1^			Over the past 2 years, my neighbourhood has got better to live and/or work in^2^			
	Overall N = 212 n (%)	Female N = 144 n (%)	Male *N* = 66 n (%)	p (sig)	Agree *N* = 65 n %	Neither agree nor disagree *N* = 56 n %	Disagree *N* = 86 n %	p (sig)
Age (category)				.296				.115
18 to 34 years	30 (14.4)	22 (15.5)	8 (12.1)		9 (13.8)	12 (21.8)	10 (11.8)	
35 to 49 years	74 (35.6)	54 (38.0)	20 (30.3)		16 (24.6)	23 (41.8)	34 (40.0)	
50 to 64 years	78 (37.5)	52 (36.6)	26 (39.4)		28 (43.1)	15 (27.3)	32 (37.6)	
65 or older	26 (12.5)	14 (9.9)	12 (18.2)		12 (18.5)	5 (9.1)	9 (10.6)	
Household income				.185				.049
Less than £20,800	53 (25.2)	38 (26.4)	15 (22.7)		17 (26.2)	10 (17.9)	26 (30.2)	
Between £20,800 & £51,999	70 (33.3)	44 (30.6)	26 (39.4)		19 (29.2)	24 (42.9)	24 (27.9)	
Greater than £52,000	29 (13.8)	17 (11.8)	12 (18.2)		14 (21.5)	8 (14.3)	6 (7.0)	
Preferred not to answer	58 (27.6)	45 (31.3)	13 (19.7)		15 (23.1)	14 (25.0)	30 (34.9)	
Number of adults in household (including self)				.097				.515
One adult	33 (15.7)	18 (12.5)	15 (22.7)		13 (20.0)	7 (12.5)	13 (15.1)	
Two adults	96 (45.7)	64 (44.4)	32 (48.5)		28 (43.1)	28 (50.0)	38 (44.2)	
Three adults	35 (16.7)	25 (17.4)	10 (15.2)		11 (16.9)	5 (8.9)	17 (19.8)	
Four or more adults	46 (21.9)	37 (25.7	9 (13.6)		13 (20.0)	16 (28.6)	18 (20.9)	
Number of children in household^4^				.778				.812
No children	100 (57.8)	71 (56.8)	29 (60.4)		27 (54.0)	26 (53.1)	43 (60.6)	
One child	31 (17.9)	22 (17.6)	9 (18.8)		10 (20.0)	7 (14.3)	14 (19.7)	
Two children	29 (16.8)	21 (16.8)	8 (16.7)		9 (18.0)	11 (22.4)	9 (12.7)	
Three or more children	13 (7.5)	11 (8.8)	2 (4.2)		4 (8.0)	5 (10.2)	5 (7.0)	
Years lived and/or worked in Stoke				.556				.002
Less than 9 years	50 (24.0)	32 (22.2)	18 (28.1)		17 (26.6)	18 (32.7)	12 (14.0)	
10 to 19 years	43 (20.7)	29 (20.1)	14 (21.9)		15 (23.4)	16 (29.1)	12 (14.0)	
20 years or more	115 (55.3)	83 (57.6)	32 (50.0)		32 (50.0)	21 (38.2)	62 (72.1)	
Connection with Stoke-on-Trent				.494				.007
Lives in Stoke-on-Trent	62 (29.5)	46 (31.9)	16 (24.2)		19 (29.2)	10 (17.9)	33 (38.4)	
Works in Stoke-on-Trent	36 (17.1)	23 (16.0)	13 (19.7)		16 (24.6)	12 (21.4)	6 (7.0)	
Both lives and works in Stoke-on-Trent	112 (53.3)	75 (52.1)	37 (56.1)		30 (46.2)	34 (60.7)	(54.7)	
Community project involved in^3^				.074				.059
*My Community Matters*	66 (34.6)	52 (40.0)	14 (23.0)		14 (23.0)	15 (29.4)	36 (46.8)	
*1000 Lives*	19 (9.9)	12 (9.2)	7 (11.5)		9 (14.8)	5 (9.8)	5 (6.5)	
*My Community Matters* & *1000 Lives*	10 (5.2)	8 (6.2)	2 (3.3)		2 (3.3)	2 (3.9)	5 (6.5)	
Other (e.g. *Neighbourhood Watch*)	98 (50.3)	58 (44.6)	38 (62.3)		36 (59.0)	29 (56.9)	31 (40.3)	

^1^Missing (n = 2); ^2^ Missing (n = 5); ^3^ Missing (*n* = 19); ^4^ Missing (*n* = 38);

Three variables influenced perceptions of whether neighbourhoods had improved as a place to live/work. Most people had lived and/or worked in their neighbourhood for at least 20 years (Table 2). Those who had lived and/or worked in their neighbourhoods for longer than 20 years were more likely than others to think their neighbourhood had not improved in the preceding 2 years, χ^2^(6) = 17,121, *p* = .002. A majority (53.3%) reported living and working in Stoke (Table 2). Those working in Stoke were more likely than those who lived in Stoke to report their neighbourhood had improved, χ^2^(4) = 14.18, *p* = .007. Most of the sample reported household income of less than £52,000, while over one quarter opted not to reveal this. Those who were in the highest household income group were significantly more likely than those in other income categories to believe their neighbourhood had improved, χ^2^(6) = 12.647, *p* = .049 (Table 2).

### Exploratory Factor Analysis and Internal Consistency Reliability

The exploratory factor analysis produced eleven distinct factors that made conceptual sense and accounted for 63.5% of the variance based on 65 of the 85 original items. The conceptual underpinnings of the new factors are described in Table 3 below.

**Table 3:** The 11 New Factors

Name of factor	Percentage of variance accounted for	Meaning of factor	Relationships to social epidemiological pathways to social well-being: networks, participation/engagement, pro-social behaviours
Collective positive effort	37.4%	Community effort	Pro-social behaviour
Participation	5.6%	Participating in group activity	Participation/engagement pathway
Celebration	4.1%	Celebrating the community	Pro-social behaviour
Social network pathway to well-being	3.1%	Comprised items representing three out of four causal paths between networks and health outcomes (Berkman and Glass 2000): support, engagement, and belonging/attachment	Network pathway
Optimism about the community	2.6%	Optimistic outlook on the community’s future	Pro-social state of mind (accompanies/leads to pro-social behaviours)
Social cohesion	2.1%	Underpinned by items linked to resolving conflict, anti-social behaviour (a cause of conflict), sharing values, being treated well, and not only looking out for oneself (Baldwin and King 2018)	Condition of the social environment for social well-being
Engagement	2.0%	Social epidemiologists describe engagement as direct results of participation (Berkman and Glass 2000), thus its well-being effects occur via the participation and engagement pathway. This factor consisted of items linked to engaging with social ties and feelings of affection, or belonging**.**	Participation/engagement pathway and/or pro-social behaviours
Safety	1.8%	Feelings of safety	Condition of the social environment for social well-being
Autonomous citizenship	1.7%	Contained items alluding to Western liberal democratic ideals of citizenship: personal liberty, free expression without harassment, fears for safety, or pressure to back a viewpoint, appreciation of community members’ contributions, and the strengthening of community spirit (Endo 2014)	Condition of the social environment for social well-being
Positive regard	1.6%	Feelings about fellow community members and mirrors a pathway between affective (emotional) attachments to community and well-being (O’Brien et al. 1994)	Affective attachments to community and well-being pathway
Low resilience	1.6%	Self-explanatory but included items on superficial friendliness and people lacking time to help others (resilience requires regular interaction with neighbours and collective problem-solving, see Baldwin and King 2018, p. 48). Residents may experience a lack of resilience until engaging in pro-social activity to rejuvenate their neighbourhood and build social resilience.	Condition of the social environment for social well-being

The 11 new factors suggest three findings. Firstly, there are six factors mirroring the social epidemiological pathways to well-being: networks, participation and engagement, and pro-social behaviours (included in the latter: collective positive effort, celebration, and optimism about the community – a pro-social state of mind). Secondly, the factor, *positive regard*, mirrors an additional pathway outlined in the community psychology literature: affective attachments to community, and well-being. Thirdly, the remaining four factors are all contemporary social conditions that can be deemed necessary pre-cursors to social well-being: social cohesion (cohesive societies and communities), safety, autonomous citizenship (individual autonomy safeguarded by the state), and resilience (current advents can lower resilience and well-being (e.g. deprivation, financial crises, climate change etc.) (Baldwin and King 2018). Without these conditions present, positive well-being may be threatened. It is interesting to note that well-being in this scale is represented by pro-social attributes that individuals can experience in group social settings and activity, but also by the right underlying social conditions.

Eight more items were removed from four factors based on conceptual considerations and improvements to internal consistency reliability with their removal. The removed items and scales included: two items (Par5 & Par6) from the Participation scale; three items (Alt4, Con3, Con5) from the Celebration scale; one item (Bel6) from the Social networking pathway to wellbeing scale, and; two items (Alt2 & Aff5) from the Autonomous citizenship scale. After removal of these items, the 11 factors contained 57 of the original 85 items. With reference to Anderson’s original NFF, two of the 11 factors resembled ‘Feelings’ (*Safety; Positive regard),* while nine factors characterised ‘Functionings’ (*Celebration, Collective positive effort, Optimism about the community; Participation; Social network pathways; Social cohesion; Autonomous citizenship).*

As shown in Table 4, internal consistency reliability, based on Cronbach alpha coefficient, was sound for each of the 11 scales. The reliability coefficients were also stable for men and women, as they were for each of the three responses to the question about whether people believed their neighbourhood had improved as a place to live and/or work in the past 2 years.

**Table 4:** Reliability coefficients overall, by sex of participant, and by respondent answers to a question about whether in the past 2 years they believed their neighbourhood had got better to live and / or work in

Factors and the items underpinning the measurement of each^1^			Sex of participant^2^		Over the past 2 years, my neighbourhood has got better to live and/or work in^3^		
	# of items	Overall (n = 212)	Female (n = 144)	Male (n = 66)	Agree (n = 65)	Neither agree nor disagree (*n* = 56)	Disagree (n = 86)
1. Collective positive effort	9	0.94	0.94	0.94	0.94	0.90	0.93
CPE1: I have noticed people in my neighbourhood helping and supporting others (*Rec6*)							
CPE2: Most of the time people in my neighbourhood try to be helpful (*Tru4*)							
CPE3: People in my neighbourhood contribute their time to help make an improvement in the neighbourhood (*Rec5*)							
CPE4: Most people in my neighbourhood try to treat me fairly (*Tru6*)							
CPE5: People in my neighbourhood would contribute their time to help with a problem in the neighbourhood (*Rec1*)							
CPE6: The people in my neighbourhood have an impact when they work together to help the neighbourhood (*Res4*)							
CPE7: People in my community pull together well when things go wrong (*Res2*)							
CPE6: People in my neighbourhood are likely to volunteer for a local cause (*Eng4*)							
CPE7*: People in my neighbourhood do not help one another (*Sup1*)							
2. Participation	3	0.75	0.74	0.79	0.75	0.63	0.76
Par1: I volunteer in person for a cause or activity in my neighbourhood (*Par4*)							
Par2: I participate in a hobby group, interest group, place of worship or religious organisation in my neighbourhood at least once per month (*Par2*)							
Par3: I participate in a range of enjoyable activities locally (*Par1*)							
3. Celebration	5	0.90	0.90	0.88	0.92	0.92	0.82
Cel1: My local community embraces the creativity of its members (*Cel2*)							
Cel2: My local community celebrates its achievements (*Cel1*)							
Cel3: My local community celebrates the diverse backgrounds of its members (*Cel6*)							
Cel4: My local community promotes shared experiences between its members (*Cel4*)							
Cel5: I appreciate the effort that people in my local community make to celebrate occasions (*Cel5*)							
4. Social network pathways	10	0.91	0.91	0.91	0.92	0.88	0.89
SNP1: I have friends, neighbours or family in my neighbourhood who I often see (*Bel7*)							
SNP2: I identify with people living in my neighbourhood (*Bel4*)							
SNP3: My neighbourhood where I live means a lot to me (*Bel5*)							
SNP4: I feel I belong in my local community (*Bel2*)							
SNP5: I communicate regularly with people in my neighbourhood about things happening in my life (*Sup2*)							
SNP6: I feel close to the people in my neighbourhood (*Bel1*)							
SNP7: I borrow things and exchange favours with people in my neighbourhood (*Sup5*)							
SNP8*: I do not have many close friends and relatives in my neighbourhood (*Sup3*)							
SNP9*: I don’t feel I have things in common with people living in my neighbourhood (*Bel3)*							
SNP10*: It’s hard to find special friends in my local community (*Aff2*)							
5. Optimism about the community	4	0.77	0.75	0.83	0.79	0.71	0.58
Opt1: The changes going on in my neighbourhood will make life better for residents (*Opt3*)							
Opt2: I feel excited when I see changes going on in my neighbourhood (*Opt4*)							
Opt3: My local community benefits from the diverse contributions of its members (*Con4*)							
Opt4*: The changes in my neighbourhood won’t make a real difference to most people’s lives (*Opt5*)							
6. Social cohesion	6	0.84	0.83	0.86	0.83	0.84	0.73
SC1: People in my local community resolve conflict in a respectful way (*Res5*)							
SC2: I have not been affected by anti-social behaviour in my neighbourhood (*Saf4*)							
SC3: People in my neighbourhood share my values (*Tru3*)							
SC4: I think that most people in my neighbourhood see a positive future here (*Opt2*)							
SC5*: People in my local community don’t treat each other well in general (*Res3*)							
SC6*: Most of the time people in my neighbourhood look out for themselves (*Tru5*)							
7. Engagement pathway	4	0.76	0.79	0.65	0.76	0.80	0.68
EP1: I make a point of learning neighbours’ names (*Eng2*)							
EP2: I keep abreast of what is happening in my neighbourhood and community (*Eng3*)							
EP3: I greet people when walking in my neighbourhood (*Eng1*)							
EP4: My local community is welcoming to newcomers (*Aff4*)							
8. Safety	5	0.85	0.85	0.85	0.78	0.86	0.82
Saf1: I feel safe walking alone in my neighbourhood after dark (*Saf1*)							
Saf2: I feel safe walking alone in my neighbourhood during the day (*Saf2*)							
Saf3*: I feel that I need to be on guard when walking in my neighbourhood (*Saf3*)							
Saf4*: I worry about crime in my neighbourhood (*Saf5*)							
Saf5*: I think that most people in my neighbourhood perceive that life here is getting worse rather than better (*Opt5*)							
9. Autonomous citizenship	5	0.85	0.85	0.85	0.85	0.78	0.82
AC1: I can express my opinions freely without fear of harassment (*Aut1*)							
AC2: I can express my opinions freely without pressure to back a particular viewpoint (*Aut2*)							
AC3*: I do not feel my opinions are listened to in neighbourhood groups (*Aut3*)							
AC4*: My local community does not appreciate contributions made by its members (*Cel3*)							
AC5*: When my community faces a challenge, it does not strengthen the community spirit (*Res5*)							
10. Positive regard	3	0.69	0.68	0.64	0.75	0.71	0.57
PR1: I treat people in my local community with respect (*Res2*)							
PR2: I think I have something valuable to give to my local community (*Con1*)							
PR3: I would provide help and support to people in my neighbourhood if they needed it (*Rec4*)							
11. Low resilience	3	0.69		0.78	0.70	0.65	0.59
LR1: My community would take a long time to get back to normal if something went wrong that affected everybody, (e.g. stormy weather, a terrorist attack, a violent crime) (*Res1*)							
LR2: My local community is friendly on the surface only (*Aff3*)							
LR3: People in my community are too busy to help others (*Alt5*)							

^1^Items show the original item number abbreviation including the flourishing dimension from which the items came in brackets; ^2^ Missing (*n* = 2); ^3^ Missing (*n* = 5);

*Reverse scored items

### Preliminary Scale Validity

Face validity and content validity were built into our study design by having content experts design the questions, which were reviewed by local community development practitioners. Evidence of construct validity was assessed initially through the conceptually coherent factors that emerged from the EFA. Convergent and divergent evidence of construct validity was then assessed from the inter-scale correlations (Table 5) as well as through the associations between each construct scale and participants answers to questions about changes in their community (Table 6).

#### Inter-Scale Correlations

As shown in Table 5, inter-scale correlations (Pearson correlation coefficients) ranged from .19 to .77. While some scales showed similarity, a majority were not highly correlated, showing support for a lack of redundancy. The only scale for which a higher score was indicative of lower community wellbeing, *Low resilience,* correlated negatively with all other scales, as expected. The strong associations between construct scales that we argue measure aspects of cohesiveness (e.g. social cohesion, collective positive effort, celebration, social network pathways, low resilience) provided some convergent evidence of construct validity.

**Table 5:** Inter-scale correlations (*n* = 212)^1^

	1	2	3	4	5	6	7	8	9	10	11
1. Collective positive effort	–	–	–	–	–	–	–	–	–	–	–
2. Participation	.31	–	–	–	–	–	–	–	–	–	–
3. Celebration	.67	.23	–	–	–	–	–	–	–	–	–
4. Social network pathway	.73	.44	.56	–	–	–	–	–	–	–	–
5. Optimism about the community	.59	.38	.56	.58	–	–	–	–	–	–	–
6. Social cohesion	.77	.19	.55	.62	.54	–	–	–	–	–	–
7. Engagement pathway	.60	.46	.49	.61	.49	.50	–	–	–	–	–
8. Safety	.64	.21	.39	.52	.45	.69	.41	–	–	–	–
9. Autonomous citizenship	.75	.30	.60	.60	.62	.68	.56	.60	–	–	–
10. Positive regard	.43	.52	.30	.40	.42	.35	.56	.27	.40	–	–
11. Low resilience	−.62	−.25	−.46	−.52	−.43	−.60	−.42	−.47	−.60	−.43	–

^1^All Pearson correlation coefficients were statistically significant *p* < .01

#### Differences in Scale Scores

To compare the mean scores of each of the 11 factors, within-subjects analyses were conducted using GLM. The total mean scores of each factor are reported in the Total column in the left-hand side of Table 6. There was an overall difference across the factors at a multivariate level, F (10, 195) = 57.93, *p* < .001. Pairwise comparisons showed the mean score on Social Cohesion was significantly (*p* < .05) lower than for all other scales except for Social Network Pathways for which there was no significant difference. At the other end of the scoring spectrum, the mean score for Positive Regard was significantly higher (p < .05) than for all other scales.

**Table 6:** Scale scores by perceptions of whether people thought their neighbourhood had got better or worse in the previous 2 years

Construct scale	Over the past 2 years, my neighbourhood has got better to live and/or work in					Over the past 2 years, my neighbourhood has got worse to live and/or work in				
	Agree (*n* = 65)	Neither agree nor disagree (n = 56)	Disagree (*n* = 86)	Total (*n* = 207)^1^	P (sig)	Agree (*n* = 81)	Neither agree nor disagree (*n* = 49)	Disagree (*n* = 72)	Total (*n* = 202)^2^	P (sig)
	M (SD)	M (SD)	M (SD)	M (SD)		M (SD)	M (SD)	M (SD)	M (SD)	
Collective positive effort	3.64 (.69)	3.45 (.56)	2.89 (.74)	3.28 (.76)	<.001	2.93 (.71)	3.22 (.65)	3.66 (.66)	3.26 (.75)	<.001
Participation	3.59 (.93)	2.98 (.75)	2.97 (.90)	3.17 (.92)	<.001	3.00 (.90)	2.94 (.86)	3.47 (.90)	3.16 (.91)	<.001
Celebration	3.47 (.85)	3.16 (.67)	2.86 (.72)	3.13 (.79)	<.001	2.85 (.69)	3.02 (.68)	3.49 (.81)	3.11 (.78)	<.001
Social network pathway	3.46 (.79)	3.10 (.63)	2.87 (.70)	3.12 (.76)	<.001	2.92 (.69)	2.90 (.67)	3.48 (.72)	3.11 (.74)	<.001
Optimism about the community	3.67 (.67)	3.24 (.52)	2.87 (.53)	3.22 (.67)	<.001	2.91 (.57)	3.17 (.53)	3.57 (.66)	3.21 (.66)	<.001
Social cohesion	3.37 (.71)	3.22 (.63)	2.59 (.60)	3.00 (.73)	<.001	2.61 (.59)	3.05 (.67)	3.41 (.66)	3.00 (.72)	<.001
Engagement pathway	3.81 (.70)	3.44 (.69)	3.21 (.68)	3.46 (.73)	<.001	3.20 (.67)	3.22 (.74)	3.88 (.56)	3.45 (.72)	<.001
Safety	3.42 (.76)	3.42 (.76)	2.72 (.75)	3.13 (.83)	<.001	2.70 (.72)	3.20 (.77)	3.54 (.74)	3.12 (.82)	<.001
Autonomous citizenship	3.54 (.74)	3.24 (.55)	2.83 (.69)	3.16 (.74)	<.001	2.89 (.65)	3.08 (.57)	3.49 (.79)	3.15 (.73)	<.001
Positive regard	4.17 (.55)	3.87 (.52)	3.77 (.53)	3.92 (.56)	<.001	3.77 (.56)	3.81 (.58)	4.15 (.47)	3.91 (.56)	<.001
Low resilience	2.64 (.76)	2.93 (.57)	3.28 (.68)	2.99 (.73)	<.001	3.25 (.65)	3.05 (.51)	2.68 (.83)	3.00 (.73)	<.001

^1^Those people who had not lived and/or worked in Stoke for at least 2 years (n = 5); ^2^ Missing as well as those people who had not lived and/or worked in Stoke for at least 2 years (*n* = 10)

#### Relationship between scale scores and perceptions of neighbourhood

To further assess preliminary evidence for convergent and divergent evidence of construct validity, participants scores on each construct scale were compared with the way they answered two statements about their perceptions of whether their neighbourhood had got better or worse, respectively, in the previous 2 years. For all scales we expected to see a linear relationship in scale scores across the three agree/neutral/disagree categories for the two neighbourhood questions. Those people who agreed with the statement that their neighbourhood had got better were expected to score higher on the NT scales (except for *Low Resilience* for which a higher score equates to lower NT) compared with those who disagreed with the statement. And the reverse was expected for the statement that their neighbourhood had got worse.

The results shown in Table 6 support the hypotheses: For all NT scales, there was a linear relationship in the expected direction with responses to both statements in each of the analyses using ANOVA with polynomial contrasts. For the statement that their neighbourhood had got better, agreement with the statement was associated with higher NT scale scores while disagreement with the statement was associated with lower scores (Collective positive effort, F(1, 204) = 47.00, *p* < .001; Participation, F(1, 204) = 17.72, p < .001; Celebration, F(1, 204) = 24.61, *p* < .001; Social network pathway, F(1, 204) = 24.81, *p* < .001; Optimism about the community, F(1, 204) = 71.78, p < .001; Social cohesion, F(1, 204) = 57.41, p < .001; Engagement pathway, F(1, 204) = 28.19, p < .001; Safety, F(1, 204) = 34.94, p < .001; Autonomous citizenship, F(1, 204) = 41.64, p < .001; Positive regard, F(1, 204) = 20.36, p < .001; Low resilience, F(1, 204) = 33.11, p < .001).

The reverse was true for the statement that the neighbourhood had got worse. Agreement with the statement was associated with lower NT scale scores while disagreement with the statement was associated with higher scores (Collective positive effort, F(1, 199) = 43.43, p < .001; Participation, F(1, 199) = 9.83, p < .001; Celebration, F(1, 199) = 28.49, p < .001; Social network pathway, F(1, 199) = 24.48, p < .001; Optimism about the community, F(1, 199) = 46.28, p < .001; Social cohesion, F(1, 199) = 61.59, p < .001; Engagement pathway, F(1, 199) = 39.92, p < .001; Safety, F(1, 199) = 48.44, p < .001; Autonomous citizenship, F(1, 199) = 28.30, p < .001; Positive regard, F(1, 199) = 19.17, p < .001; Low resilience, F(1, 199) = 26.17, p < .001). These results provide some preliminary evidence of construct validity.

### Neighbourhood Thriving Scale Scores by Participant Characteristics

Overall, the sample of pro-social volunteers did not endorse their neighbourhood as being a better place to live and/or work in compared with 2 years prior.

#### Age and Neighbourhood Thriving

ANOVA with linear (polynomial) contrasts were used to test the relationship between the four age categories and each of the scale scores. As shown in Table 7, three of the scales showed a linear trend with age: *Participation*, *Social Network Pathway*, and *Engagement Pathway*. For each of these scales the significant trend was in the direction of neighbourhood thriving being positively (directly) related with age; older people experiencing neighbourhood thriving to a greater extent than younger people.

**Table 7:** Neighbourhood thriving scale scores by participant characteristics (N = 212)

		Neighbourhood thriving scales										
	N	Collective positive effort	Participation	Celebration	Social network pathways	Optimism	Social cohesion	Engagement pathways	Safety	Autonomous citizenship	Positive regard	Low resilience
		M (SD)	M (SD)	M (SD)	M (SD)	M (SD)	M (SD)	M (SD)	M (SD)	M (SD)	M (SD)	M (SD)
Age (category) (Total) 18 to 34 years 35 to 49 years 50 to 64 years 65 or older	210	3.28 (.75)	3.17 (.91)	3.12 (.79)	3.12 (.75)	3.22 (.67)	3.02 (.73)	3.48 (.73)	3.13 (.82)	3.16 (.73)	3.93 (.56)	2.97 (.74)
	31 74 79 26	3.21 (.90) 3.28 (.64) 3.30 (.80) 3.34 (.76)	2.86 (.88) 3.09 (.82) 3.34 (.97) 3.28 (.93)	3.19 (.98) 3.11 (.67) 3.04 (.85) 3.31 (.66)	2.86 (.94) 3.17 (.61) 3.10 (.80) 3.38 (.66)	3.10 (.74) 3.25 (.57) 3.21 (.76) 3.31 (.52)	2.97 (.77) 3.04 (.60) 2.98 (.84) 3.12 (.70)	3.20 (.75) 3.45 (.74) 3.55 (.70) 3.63 (.70)	3.01 (.93) 3.21 (.75) 3.10 (.87) 3.17 (.76)	3.13 (.78) 3.14 (.62) 3.21 (.79) 3.15 (.82)	3.88 (.59) 3.89 (.55) 4.00 (.56) 3.88 (.58)	3.00 (.68) 3.03 (.65) 2.89 (.77) 3.03 (.94)
F statistic (Linear) (df = 1, 206) (sig)		.45	6.08*	.025	4.06*	.66	.18	6.13*	.08	.12	.47	.22
Household income (Total) ^5^ Less than £20,800 Between £20,800 & £51,999 Greater than £52,000	152	3.42 (.76)	3.36 (.91)	3.20 (.85)	3.21 (.78)	3.29 (.71)	3.09 (.71)	3.65 (.64)	3.28 (.86)	3.32 (.72)	4.07 (.49)	2.84 (.75)
	53 70 29	3.09 (.87) 3.49 (.63) 3.84 (.56)	3.50 (.94) 3.24 (.87) 3.40 (.95)	2.99 (.94) 3.22 (.79) 3.52 (.72)	3.04 (.87) 3.21 (.71) 3.54 (.65)	3.08 (.79) 3.36 (.62) 3.49 (.67)	2.78 (.86) 3.19 (.69) 3.43 (.62)	3.49 (.65) 3.68 (.62) 3.91 (.59)	3.04 (.83) 3.30 (.90) 3.68 (.67)	3.08 (.80) 3.36 (.68) 3.69 (.49)	4.03 (.48) 4.08 (.50) 4.10 (.49)	3.14 (.74) 2.70 (.76) 2.61 (.58)
F statistic (Linear) (df = 1151) (sig)		22.53***	.56	7.58**	7.85**	7.85**	16.23***	8.82**	11.03**	14.91***	.46	12.78***
Years lived in Stoke (Total) Less than 9 years 10 to 19 years 20 years or more	210	3.28 (.75)	3.17 (.92)	3.11 (.75)	3.11 (.75)	3.22 (.67)	3.01 (.73)	3.46 (.72)	3.13 (.82)	3.16 (.73)	3.92 (.56)	2.97 (.74)
	51 43 116	3.31 (.82) 3.54 (.65) 3.16 (.73)	2.96 (.87) 3.39 (.95) 3.18 (.91)	3.21 (.88) 3.33 (.71) 2.98 (.76)	2.92 (.80) 3.25 (.67) 3.15 (.75)	3.25 (.64) 3.37 (.60) 3.15 (.69)	3.15 (.76) 3.16 (.70) 2.89 (.71)	3.47 (.81) 3.59 (.60) 3.41 (.73)	3.19 (.89) 3.41 (.78) 3.00 (.79)	3.22 (.68) 3.42 (.64) 3.03 (.76)	3.92 (.60) 4.04 (.51) 3.88 (.57)	2.86 (.64) 2.82 (.66) 3.08 (.79)
F statistic (B/W group) (df = 2, 207) (sig)		4.15*	2.59	3.71*	2.65	1.77	3.34*	1.04	4.31*	4.76*	1.30	2.82
Connection with Stoke-on-Trent Lives in Stoke-on-Trent Works in Stoke-on-Trent Lives and works in Stoke-on-Trent	212	3.28 (.75)	3.17 (.91)	3.12 (.79)	3.12 (.75)	3.22 (.67)	3.01 (.73)	3.47 (.73)	3.13 (.82)	3.16 (.73)	3.92 (.56)	2.97 (.73)
	63 36 113	3.00 (.85) 3.59 (.61) 3.34 (.68)	3.29 (.89) 3.06 (.88) 3.13 (.93)	2.92 (.81) 3.34 (.71) 3.16 (.78)	3.03 (.76) 3.18 (.67) 3.15 (.77)	3.02 (.67) 3.52 (.52) 3.23 (.67)	2.75 (.75) 3.29 (.63) 3.08 (.71)	3.32 (.77) 3.62 (.74) 3.51 (.69)	2.89 (.78) 3.33 (.75) 3.21 (.84)	2.86 (.80) 3.48 (.59) 3.22 (.67)	3.88 (.61) 3.93 (.59) 3.95 (.53)	3.23 (.67) 2.71 (.61) 2.91 (.76)
F statistic (B/W group) (df = 2, 209) (sig)		8.23*	.84	3.67*	.60	6.98**	7.67**	2.31	4.48*	10.12***	.30	6.97**

^1^Missing (n = 2); ^2^ Missing (n = 5); ^3^ Missing (n = 19); ^4^ Missing (n = 38); ^5^ The last category – those who did not want to answer the question – was removed to enable linear analysis

*p < .05; **p < .01; ***p < .001

#### Household Income and Neighbourhood Thriving

Participants who indicated their preference not to answer the question about their household income (*n* = 58; 27.6%) were excluded from this analysis so that an ANOVA with linear (polynomial) contrasts could be conducted. These analyses, which included three household income categories, showed a significant trend with 9 of the 11 neighbourhood thriving scales: *Collective Positive Effort*, *Celebration, Social Network Pathway*, *Optimism, Social Cohesion, Engagement Pathway, Safety, Autonomous Citizenship,* and *Low Resilience.* Each significant trend was in the direction of a positive (direct) relationship between income and NT: the higher the income the greater sense and experience of NT.

#### Years Lived and/or Worked in Stoke and Neighbourhood Thriving

Time lived and/or worked in Stoke (analysed as three categories) were analysed against the NT scales using ANOVA for between-subjects effects as such analyses explained the data more accurately than linear trend. Scores on five of the 11 scales were associated with time lived in Stoke: *Collective Positive Effort*, *Celebration, Social Cohesion, Safety,* and *Autonomous Citizenship.* In each of these associations, except for *Social Cohesion,* those who had lived and/or worked in Stoke for 10 to 19 years showed higher levels of NT compared with participants who had lived and/or worked there for less than 9 years or greater than or equal to 20 years. For *Social Cohesion*, there was greater NT among those who had lived and/or worked in Stoke for 10 to 19 years compared with the people who had lived in Stoke for 20 years or more.

#### Presence in Stoke

The type of presence people had in Stoke (analysed as three categories: living; working; living and working in Stoke) were analysed against the NT scales using ANOVA for between-subjects effects. Scores on seven of the 11 scales were associated with connection with Stoke: *Collective Positive Effort*, *Celebration, Optimism, Social Cohesion, Safety, Autonomous Citizenship, and Low Resilience.* In each of these associations, those who worked in Stoke only (i.e. did not live in Stoke) showed higher levels of NT compared with participants living there, and those who both lived and worked in Stoke.

## Discussion

Our first trial of the NTF resulted in useable questionnaire data from 212 participants. Exploratory factor analysis (EFA) produced 11 conceptually coherent factors that were further refined through the omission of items that were not contributing conceptually or to internal consistency reliability. All scales showed satisfactory internal consistency reliability across the entire sample as well as separately for men and women and for all three responses to the question about whether participants thought their neighbourhood had improved over the previous 2 years.

While face and content validity were built into the design of the scales, construct validation was limited to preliminary evidence only through inter-scale correlations and the relationships of the scales to two questions which asked participants whether they thought their neighbourhood had got better or worse, respectively, in the previous 2 years. Inferring construct validity from these analyses is limited because the variables that were used to validate the scales against, were broad areas and not specific to each scale. As such, these associations are only able to confirm that the scales were associated with other factors in predictable ways. Additional research is needed to validate individual scales against other known validated scales that are measuring similar constructs. Until that happens and the body of evidence for construct validation builds, there must be caution in making inferences when using the scales. Other validation work that is needed is in showing whether the scales can be used to predict (at least correlate with) behaviours consistent with a person’s scores on each construct, and also examining whether a total NT score is predictive of behaviours. It will also be important for future studies to apply and validate the scales in populations that are comprised of people other than pro-social volunteers, particularly in random samples of local communities. Until that happens, we cannot be sure of the stability of the scale structure for non pro-social populations. As such, caution should be exercised when seeking to draw conclusions from the results of administering the scale to non pro-social populations. Notwithstanding the need for further validation studies, what do the new scales tell us about NT in our sample?

Caution is required in making any claims about levels of NT until further validation and norm scores are established. However, since all scale items were measured on a 5-point likert scale from 1 to 5, we offer some broad observations based on mean scores for the scales. With a mid-point of 3 on the 5-point Likert scale, and based on higher scores for each of the scales (except for *Low Resilience*) indicating higher levels of NT, we can observe that the mean of each of the scales was equal to or above the mid-point, likely indicative of NT. The *Social Cohesion* scale was scored the lowest of all the scales which suggests many in the sample perceived social difference in their neighbourhoods as being somewhat problematic rather than as a richness to celebrate. The Celebration scale was also one of the lowest scored scales, albeit above the mid-point, which suggests a relative absence of activities and events that recognise and celebrate community achievements. At the opposite end, the *Positive Regard* scale was scored highest of all the scales which indicates that people may have experienced a positive outlook towards others in their neighbourhood, perhaps because they are intimately involved in revitalisation activities.

As for who appears to be experiencing the greatest levels of NT, the results indicate it is positively (linearly) associated with age and income: older people and those with higher household incomes. Also, those who had lived and/or worked in Stoke for 10–19 years showed higher levels of aspects of NT compared with those who had been living or working there for shorter or greater periods of time. The type of involvement was also important, with those who work in Stoke showing higher levels of aspects of NT compared with those people who lived, some of whom also worked, in Stoke. These results are consistent with findings from the ‘ageing in place’ literature (e.g. Young et al. 2004) which often show that older residents are less likely to move from their long-term place of residence and feel attached to it, which may help explain higher levels of well-being. Similarly, wealthier people are less likely to experience adverse living circumstances and associated stress, therefore demonstrate higher levels of well-being (Wilkinson and Marmot 2003). Those not living but just working in Stoke could be hypothesised as more likely to live in pleasant surrounding rural areas. Likewise, those in the middle ‘length of residence’ category had lived in Stoke long enough to have evolved networks and experienced residential stability, but not so long as to become jaded with the deprived environment.

It may surprise some readers that the sample of pro-social volunteers in this study did not confirm their neighbourhood had become a better place to live and/or work in over the previous 2 years. There are some plausible reasons for this finding. One explanation is that people who volunteer to try and improve their social environment are motivated by observing that their neighbourhoods could become more pleasant places to live and work. Without that observation and viewpoint, it is unlikely people would be motivated to volunteer for such roles. As such, one may conclude that a sample of pro-social volunteers are likely to be the harshest critics of their social environments as they are the ones who observed that change was necessary and chose to act upon that realisation. This is another reason why it is important to sample from a broader cross-section of the community to gauge wider-held community perceptions of changes in the neighbourhood.

## Policy Recommendations

Since the current study did not set out to evaluate the two community development projects that were commissioned by Stoke-on-Trent City Council Public Health Department in 2012 to reduce health inequalities through social action, and because the current sample comprised pro-social volunteers and not a broad cross-section of the community, we are unable to ascertain the specific benefits of these programmes to their communities. However, the 11-factor NT scale that emerged from our research revealed social conditions key to social well-being in the local social environment. Future community development programmes should aim to address these conditions: the first three of which can be cultivated through local action: social cohesion (through structured mixing of people from different backgrounds in collaborative activities), safety (through design efforts to transform urban environments with poor street lighting, poor traffic control, and no eyes on the street, and the targeted reduction both crime and violence, and their risk factors), and resilience (through cultivating social capital, social cohesion, well-being and the eradication of inequalities). Autonomous citizenship is both a national and local benefit that individual volunteers may experience feelings of it if, during civic action, they can express themselves freely, their fears for safety are allayed, appreciation is shown for volunteers' input, and a feeling of community spirit is garnered.

## Conclusions

In this paper we have taken steps towards developing a neighbourhood thriving scale underpinned by various sub-scales. Each sub-scale showed satisfactory internal consistency reliability and early signs of construct validity. More validation work is needed on these scales, particularly with non pro-social volunteers, before they can be used with confidence to measure the 11 dimensions identified in the study. When these measures were applied to a sample of pro-social community volunteers in Stoke, we observed the highest scale score for *Positive Regard* and the lowest scale score for *Celebration*. Gauging levels of NT through comparison with other places will be made possible once further validation and norms are established. We also observed a positive relationship between age and income with neighbourhood thriving, suggestive that NT may not be experienced equally and to the same extent by all Stoke residents. The scale may develop into a useful tool for evaluating the success of community projects when administered pre and post project implementation. It may also be useful for gauging which members of a community are experiencing low levels of NT and to tailor the design of projects at different groups of community members and their needs.

## Electronic supplementary material

ESM 1 (DOCX 35 kb)
