# Supplementary material for: Measuring Well-Being: Trial of the Neighbourhood Thriving Scale for Social Well-Being Among Pro-Social Individuals
Source: Int J Community Wellbeing. 2020 Jun 10;3(3):361–90. doi: 10.1007/s42413-020-00067-6 (PMC7286207; doi:10.1007/s42413-020-00067-6)
Supplement: Supplementary file 1 — (DOCX 35 kb) [file 42413_2020_67_MOESM1_ESM.docx]

**Appendix A:**

**Initial scale item statements & source of item development**

1. **Belonging (7 items)**

**Thinking about your experiences in your neighbourhood and community, to what extent do you agree or disagree with each of the following statements?**

Bel1: I feel close to the people in my neighbourhood
Bel2: I feel I belong in my local community
Bel3: I don’t feel I have things in common with people living in my neighbourhood
Bel4: I identify with people living in my neighbourhood
Bel5: My neighbourhood where I live means a lot to me
Bel6: I don’t feel I belong in my neighbourhood
Bel7: I have friends, neighbours or family in my neighbourhood who I often see

Items Bel1 to Bel3 were adapted from Rawstorne’s unpublished scales on belonging within Aboriginal and gay communities in Australia. Bel5 was adapted from an item measuring emotional attachment to place (‘place attachment’) in an environmental psychology questionnaire: ‘X [*name* *place*] means a lot to me’ (Williams and Vaske 2003, p.835). Bel7 was adapted from the item ‘Frequency of seeing/speaking to relatives/friends/neighbours’ used to measure the network component of social capital in a review by the UK government Office for National Statistics (ONS) (Foxton and Jones 2011, p.4).

1. **Respect (5 items)**

**Thinking about your experiences in your neighbourhood and community, to what extent do you agree or disagree with each of the following statements?**

Res1: People in my local community treat me with respect
Res2: I treat people in my local community with respect
Res3: People in my local community don’t treat each other well in general
Res4: I am comfortable living alongside people from different backgrounds in my neighbourhood
Res5: People in my local community resolve conflict in a respectful way

Items Res1 and Res2 were adapted from the ESS (Huppert et al. 2012). Res3 to Res5 were variations on a question from the *Citizenship Survey* (2010-11): ‘To what extent do you agree or disagree that this local area is a place where people from different backgrounds get on well together?’

The third question (Res3) varied only by omitting the word ‘community’ as it was applicable to the neighbourhood context only. The same pattern was followed with the remaining questions whereby the geographic (neighbourhood) or social (community) context or both were inserted, as applicable.

1. **Trust (6 items)**

**Thinking about your experiences of your neighbourhood, to what extent do you agree or disagree with each of the following statements?**

Tru1: Most people in my neighbourhood can be trusted
Tru2: Most people in my neighbourhood would try to take advantage of me if they got the chance
Tru3: People in my neighbourhood share my values
Tru4: Most of the time people in my neighbourhood try to be helpful
Tru5: Most of the time people in my neighbourhood look out for themselves
Tru6: Most people in my neighbourhood try to treat me fairly

Tru1 was adapted from similar questions in the *Citizenship Survey* (2010-11) and the ESS (Huppert et al. 2012). Tru2-Tru4 were adapted from the ESS (2012).

1. **Support (5 items)**

**Thinking about your experiences of life in your neighbourhood and community, to what extent do you agree or disagree with each of the following statements?**

Sup1: People in my neighbourhood do not help one another
Sup2: I communicate regularly with people in my neighbourhood about things happening in my life
Sup3: I do not have many close friends and relatives in my neighbourhood
Sup4: I have a spouse or partner, family member, friend or neighbour living in my neighbourhood whom I can rely on a lot if I have a serious problem
Sup5: I borrow things and exchange favours with people in my neighbourhood

Item Sup1 was adapted from an item measuring social networks and social support in the ONS review of social capital: ‘Exchange of help’ (Foxton and Jones 2011, p.4). Sup2 was adapted from a definition of social support nominated by Cobb (1976, p.300), that an individual ‘belongs to a social network of communication and mutual obligation.’ Sup4 and Sup5 were adapted from the UK survey, ‘Understanding Society: UK Household Longitudinal Study 2013-2014’.

1. **Safety (5 items)**

**Thinking about your experiences of living in and moving around your neighbourhood, to what extent do you agree or disagree with each of the following statements?**

Saf1: I feel safe walking alone in my neighbourhood after dark
Saf2: I feel safe walking alone in my neighbourhood during the day
Saf3: I feel that I need to be on guard when walking in my neighbourhood
Saf4: I have not been affected by anti-social behaviour in my neighbourhood
Saf5: I worry about crime in my neighbourhood

Items Saf1 and Saf2 were adapted from the *Citizenship Survey*. Saf4 and Saf5 were statements that were made by many local residents in the first author’s PhD ethnographic study (Baldwin 2012) of Swindon, a comparable post-industrial town in England.

1. **Reciprocity (6 items)**

**Thinking about your experiences of life in your neighbourhood, to what extent do you agree or disagree with each of the following statements?**

Rec1: People in my neighbourhood would contribute their time to help with a problem in the neighbourhood
Rec2: I would expect to receive help and support from people in my neighbourhood if I needed it
Rec3: I avoid people in my neighbourhood because they expect too much of my time
Rec4: I would provide help and support to people in my neighbourhood if they needed it
Rec5: People in my neighbourhood contribute their time to help make an improvement in the neighbourhood
Rec6: I have noticed people in my neighbourhood helping and supporting others

Item Rec2 was again based on the item ‘Exchange of help’ from the ONS social capital review (Foxton and Jones 2011, p.4).

1. **Celebration (6 items)**

**Thinking about your experiences of the community in your neighbourhood, to what extent do you agree or disagree with each of the following statements?**

Cel1: My local community celebrates its achievements
Cel2: My local community embraces the creativity of its members
Cel3: My local community does not appreciate contributions made by its members
Cel4: My local community promotes shared experiences between its members
Cel5: I appreciate the effort that people in my local community make to celebrate occasions
Cel6: My local community celebrates the diverse backgrounds of its members

Items A-C are based on items devised by third author (in Anderson and Baldwin 2017) in response to a focus group item about ‘creativity’ in Rogers (2005, p.118): ‘Does your community encourage imagination, boldness, inventiveness, and curiosity?’.

1. **Engagement (5 items)**

**Thinking about your experiences of the community in your neighbourhood,** **to what extent do you agree or disagree with each of the following statements?**

Eng1: I greet people when walking in my neighbourhood
Eng2: I make a point of learning neighbours’ names
Eng3: I keep abreast of what is happening in my neighbourhood and community
Eng4: People in my neighbourhood are likely to volunteer for a local cause
Eng5: People in my neighbourhood are likely to take action on an important local issue

Items Eng1 to Eng4 were devised by the third author (Anderson) (in Anderson and Baldwin 2017).

1. **Autonomy (5 items)**

**Thinking about your experiences of participating in neighbourhood decision-making, to what extent do you agree or disagree with each of the following statements?**

Aut1: I can express my opinions freely without fear of harassment
Aut2: I can express my opinions freely without pressure to back a particular viewpoint
Aut3: I do not feel my opinions are listened to in neighbourhood groups
Aut4: I can influence decisions affecting my neighbourhood
Aut5: People in my neighbourhood are free to choose whether to participate in neighbourhood activities

Items Aut1, Aut2 and Aut5 draw from a focus group item pertaining to ‘freedom’ (Rogers 2005, p.10): ‘Does your community encourage independent thought and action?’ Aut4 is adapted from an item used to measure the civic participation component of social capital: ‘Perceptions of ability to influence events’ in the ONS review (Foxton and Jones 2011, p.4).

1. **Resilience (6 items)**

**Thinking about how well your community responds to issues and problems together rather than individually, to what extent do you agree or disagree with each of the following statements?**

Res1: My community would take a long time to get back to normal if something went wrong that affected everybody, e.g. stormy weather, a terrorist attack, a violent crime
Res2: People in my community pull together well when things go wrong
Res3: The people in my neighbourhood aren't good at working together to achieve things
Res4: The people in my neighbourhood have an impact when they work together to help the neighbourhood
Res5: When my community faces a challenge, it does not strengthen the community spirit
Res6: My community is stronger because there are people from different backgrounds living in the neighbourhood

1. **Altruism (5 items)**

**Thinking about your experiences of life in your neighbourhood and community, to what extent do you agree or disagree with each of the following statements?**

Alt1: People in my neighbourhood don’t expect help in return if they help someone living in the neighbourhood
Alt2: People around here only think about themselves
Alt3: People in my neighbourhood would stop to help a stranger in trouble
Alt4: My local community raises money for good causes
Alt5: People in my community are too busy to help others

Item Res1 in questions 11-13 is adapted in each case from items devised by the third author (Anderson) (in Anderson and Baldwin 2017).

1. **Contribution (5 items)**

**Thinking about your experiences of life in your neighbourhood and community, to what extent do you agree or disagree with each of the following statements?**

Con1: I think I have something valuable to give to my local community
Con2: My local community does not value what I could give to neighbourhood projects
Con3: My local community values the contributions of its members
Con4: My local community benefits from the diverse contributions of its members
Con5: My local community does not actively encourage local people to offer their skills and ideas

1. **Optimism (5 items)**

**Thinking about your experiences of life in your neighbourhood, to what extent do you agree or disagree with each the following statements?**

Opt1: I think that most people in my neighbourhood perceive that life here is getting worse rather than better
Opt2: I think that most people in my neighbourhood see a positive future here
Opt3: The changes going on in my neighbourhood will make life better for residents
Opt4: I feel excited when I see changes going on in my neighbourhood
Opt5: The changes in my neighbourhood won’t make a real difference to most people’s lives

1. **Participation (7 items)**

**Thinking about your experiences of life in your neighbourhood and community, to what extent do you agree or disagree with each the following statements?**

Par1: I participate in a range of enjoyable activities locally
Par2: I participate in a hobby group, interest group, place of worship or religious organisation in my neighbourhood at least once per month
Par3: I prefer my own company to attending groups in my neighbourhood
Par4: I volunteer in person for a cause or activity in my neighbourhood
Par5: The same people seem to do everything in my neighbourhood
Par6: I participate in online communities focused on my neighbourhood
Par7: I’m not interested in activities or groups in my neighbourhood

Item Par1 is adapted from an item devised by the third author (Anderson) (in Anderson and Baldwin 2017). Par2 is adapted from an item used to measure the social participation component of social capital in the ONS review: ‘Number of cultural, leisure, social groups belonged to and frequency and intensity of involvement’ (Foxton and Jones 2011, p.4).

1. **Affection (5 items)**

**Thinking about your experiences of life in your local community, to what extent do you agree or disagree with each the following statements?**

Aff1: My local community is friendly
Aff2: It’s hard to find special friends in my local community
Aff3: My local community is friendly on the surface only
Aff4: My local community is welcoming to newcomers
Aff5: Friendliness in my local community depends on your social standing

Item Aff1 is adapted from a focus group item about ‘affection’: ‘How welcoming and friendly is your community?’ from Rogers (2005, p. 119). Aff2, Aff3, Aff4 and Aff5 are based on focus group participants’ responses to that question.

**Appendix B:**

**Final scale dimensions**

***Instructions:* Thinking about your experiences in your neighbourhood and community, to what extent do you disagree or agree with each of the following statements? There are no right or wrong answers. For each statement, please select one option only to indicate if you strongly disagree, disagree, neither disagree or agree, agree or strongly agree. If you are unsure, please give your best guess.**

| Strongly disagree  (1) | Disagree  (2) | Neither disagree nor agree  (3) | Agree  (4) | Strongly agree  (5) |
| --- | --- | --- | --- | --- |

1. **Collective positive effort (9 items)**

CPE1: I have noticed people in my neighbourhood helping and supporting others (*Rec6*)
CPE2: Most of the time people in my neighbourhood try to be helpful (*Tru4*)
CPE3: People in my neighbourhood contribute their time to help make an improvement in the neighbourhood (*Rec5*)
CPE4: Most people in my neighbourhood try to treat me fairly (*Tru6*)
CPE5: People in my neighbourhood would contribute their time to help with a problem in the neighbourhood (*Rec1*)
CPE6: The people in my neighbourhood have an impact when they work together to help the neighbourhood (*Res4*)
CPE7: People in my community pull together well when things go wrong (*Res2*)

CPE8: People in my neighbourhood are likely to volunteer for a local cause (*Eng4*)
CPE9*: People in my neighbourhood do not help one another (*Sup1*)

1. **Participation (3 items)**

Par1: I volunteer in person for a cause or activity in my neighbourhood (*Par4*)
Par2: I participate in a hobby group, interest group, place of worship or religious organisation in my neighbourhood at least once per month (*Par2*)
Par3: I participate in a range of enjoyable activities locally (*Par1*)

1. **Celebration (5 items)**

Cel1: My local community embraces the creativity of its members (*Cel2*)
Cel2: My local community celebrates its achievements (*Cel1*)
Cel3: My local community celebrates the diverse backgrounds of its members (*Cel6*)
Cel4: My local community promotes shared experiences between its members (*Cel4*)
Cel5: I appreciate the effort that people in my local community make to celebrate occasions (*Cel5*)

1. **Social network pathways (10 items)**

SNP1: I have friends, neighbours or family in my neighbourhood who I often see (*Bel7*)
SNP2: I identify with people living in my neighbourhood (*Bel4*)
SNP3: My neighbourhood where I live means a lot to me (*Bel5*)

SNP4: I feel I belong in my local community (*Bel2*)
SNP5: I communicate regularly with people in my neighbourhood about things happening in my life (*Sup2*)
SNP6: I feel close to the people in my neighbourhood (*Bel1*)
SNP7: I borrow things and exchange favours with people in my neighbourhood (*Sup5*)

SNP8*: I do not have many close friends and relatives in my neighbourhood (*Sup3*)
SNP9*: I don’t feel I have things in common with people living in my neighbourhood (*Bel3)*
SNP10*: It’s hard to find special friends in my local community (*Aff2*)

1. **Optimism about the community (4 items)**

Opt1: The changes going on in my neighbourhood will make life better for residents (*Opt3*)
Opt2: I feel excited when I see changes going on in my neighbourhood (*Opt4*)
Opt3: My local community benefits from the diverse contributions of its members (*Con4*)
Opt4*: The changes in my neighbourhood won’t make a real difference to most people’s lives (*Opt5*)

1. **Social cohesion (6 items)**

SC1: People in my local community resolve conflict in a respectful way (*Res5*)
SC2: I have not been affected by anti-social behaviour in my neighbourhood (*Saf4*)
SC3: People in my neighbourhood share my values (*Tru3*)
SC4: I think that most people in my neighbourhood see a positive future here (*Opt2*)
SC5*: People in my local community don’t treat each other well in general (*Res3*)
SC6*: Most of the time people in my neighbourhood look out for themselves (*Tru5*)

1. **Engagement pathway (4 items)**

EP1: I make a point of learning neighbours’ names (*Eng2*)
EP2: I keep abreast of what is happening in my neighbourhood and community (*Eng3*)
EP3: I greet people when walking in my neighbourhood (*Eng1*)
EP4: My local community is welcoming to newcomers (*Aff4*)

1. **Safety (5 items)**

Saf1: I feel safe walking alone in my neighbourhood after dark (*Saf1*)
Saf2: I feel safe walking alone in my neighbourhood during the day (*Saf2*)
Saf3*: I feel that I need to be on guard when walking in my neighbourhood (*Saf3*)
Saf4*: I worry about crime in my neighbourhood (*Saf5*)
Saf5*: I think that most people in my neighbourhood perceive that life here is getting worse rather than better (*Opt5*)

1. **Autonomous citizenship (5 items)**

AC1: I can express my opinions freely without fear of harassment (*Aut1*)
AC2: I can express my opinions freely without pressure to back a particular viewpoint (*Aut2*)
AC3*: I do not feel my opinions are listened to in neighbourhood groups (*Aut3*)
AC4*: My local community does not appreciate contributions made by its members (*Cel3*)
AC5*: When my community faces a challenge, it does not strengthen the community spirit (*Res5*)

1. **Positive regard (3 items)**

PR1: I treat people in my local community with respect (*Res2*)
PR2: I think I have something valuable to give to my local community (*Con1*)
PR3: I would provide help and support to people in my neighbourhood if they needed it (*Rec4*)

1. **Low resilience (3 items)**

LR1: My community would take a long time to get back to normal if something went wrong that affected everybody, e.g. stormy weather, a terrorist attack, a violent crime (*Res1*)
LR2: My local community is friendly on the surface only (*Aff3*)
LR3: People in my community are too busy to help others (*Alt5*)

Note for those who use this scale:

Higher scores on all scales except for *Low Resilience* equate to greater levels of neighbourhood flourishing. Higher scores on *Low Resilience* equate to lower levels of neighbourhood flourishing. The *Low Resilience* scale negatively correlates with the other 10 scales.

Labels in brackets after each item refer to the original item label for reference only and are for the benefit of readers and those who will be administering the scales. These labels in brackets ought to be omitted when administering the scales.

We have suggested here that a likert scale be used from 1 Strongly disagree to 5 Strongly agree which is the opposite direction to how it was used in the current study, which was 1 Strongly agree to 5 Strongly disagree. This suggested change was precipitated by the number of reverse scoring of items required in the current study which will be minimised by changing the direction of the likert scale. References to reverse scoring items in Appendix B (see * below) are based on using a likert scale from from 1 Strongly disagree to 5 Strongly agree.

*Reverse score these items so that a higher score indicates greater levels of neighbourhood flourishing.
